# Pain in Parkinson's disease: a neuroanatomy-based approach

**DOI:** 10.1093/braincomms/fcae210

**Published:** 2024-06-18

**Authors:** Domiziana Nardelli, Francesco Gambioli, Maria Ilenia De Bartolo, Romina Mancinelli, Francesca Biagioni, Simone Carotti, Emma Falato, Giorgio Leodori, Stefano Puglisi-Allegra, Giorgio Vivacqua, Francesco Fornai

**Affiliations:** Laboratory of Microscopic and Ultrastructural Anatomy, Campus Biomedico University of Roma, Rome 00128, Italy; Laboratory of Microscopic and Ultrastructural Anatomy, Campus Biomedico University of Roma, Rome 00128, Italy; IRCCS Neuromed, Pozzilli, IS 86077, Italy; Department of Anatomical, Histological, Forensic Medicine and Orthopedic Sciences, Sapienza University of Roma, Rome 00161, Italy; IRCCS Neuromed, Pozzilli, IS 86077, Italy; Laboratory of Microscopic and Ultrastructural Anatomy, Campus Biomedico University of Roma, Rome 00128, Italy; Laboratory of Microscopic and Ultrastructural Anatomy, Campus Biomedico University of Roma, Rome 00128, Italy; IRCCS Neuromed, Pozzilli, IS 86077, Italy; Department of Human Neuroscience, Sapienza University of Roma, Rome 00185, Italy; IRCCS Neuromed, Pozzilli, IS 86077, Italy; Laboratory of Microscopic and Ultrastructural Anatomy, Campus Biomedico University of Roma, Rome 00128, Italy; IRCCS Neuromed, Pozzilli, IS 86077, Italy; Department of Experimental Morphology and Applied Biology, University of Pisa, Pisa 56122, Italy

**Keywords:** pain, Parkinson’s disease, dopamine, limbic system, alpha-synuclein

## Abstract

Parkinson's disease is a progressive neurodegenerative disorder characterized by the deposition of misfolded alpha-synuclein in different regions of the central and peripheral nervous system. Motor impairment represents the signature clinical expression of Parkinson's disease. Nevertheless, non-motor symptoms are invariably present at different stages of the disease and constitute an important therapeutic challenge with a high impact for the patients’ quality of life. Among non-motor symptoms, pain is frequently experienced by patients, being present in a range of 24–85% of Parkinson's disease population. Moreover, in more than 5% of patients, pain represents the first clinical manifestation, preceding by decades the exordium of motor symptoms. Pain implies a complex biopsychosocial experience with a downstream complex anatomical network involved in pain perception, modulation, and processing. Interestingly, all the anatomical areas involved in pain network can be affected by a-synuclein pathology, suggesting that pathophysiology of pain in Parkinson's disease encompasses a ‘pain spectrum’, involving different anatomical and neurochemical substrates. Here the various anatomical sites recruited in pain perception, modulation and processing are discussed, highlighting the consequences of their possible degeneration in course of Parkinson's disease. Starting from peripheral small fibres neuropathy and pathological alterations at the level of the posterior laminae of the spinal cord, we then describe the multifaceted role of noradrenaline and dopamine loss in driving dysregulated pain perception. Finally, we focus on the possible role of the intertwined circuits between amygdala, nucleus accumbens and habenula in determining the psycho-emotional, autonomic and cognitive experience of pain in Parkinson's disease. This narrative review provides the first anatomically driven comprehension of pain in Parkinson's disease, aiming at fostering new insights for personalized clinical diagnosis and therapeutic interventions.

## Teaser

Nardelli *et al*., analysed the different anatomical pathways underlaying nociceptive, neuropathic and nociplastic pain in Parkinson's disease, proposing a neuroanatomy-based pain classification, aiming at fostering new insight for personalized clinical diagnosis and therapeutic interventions.

## Graphical Abstract

**Graphical Abstract:**
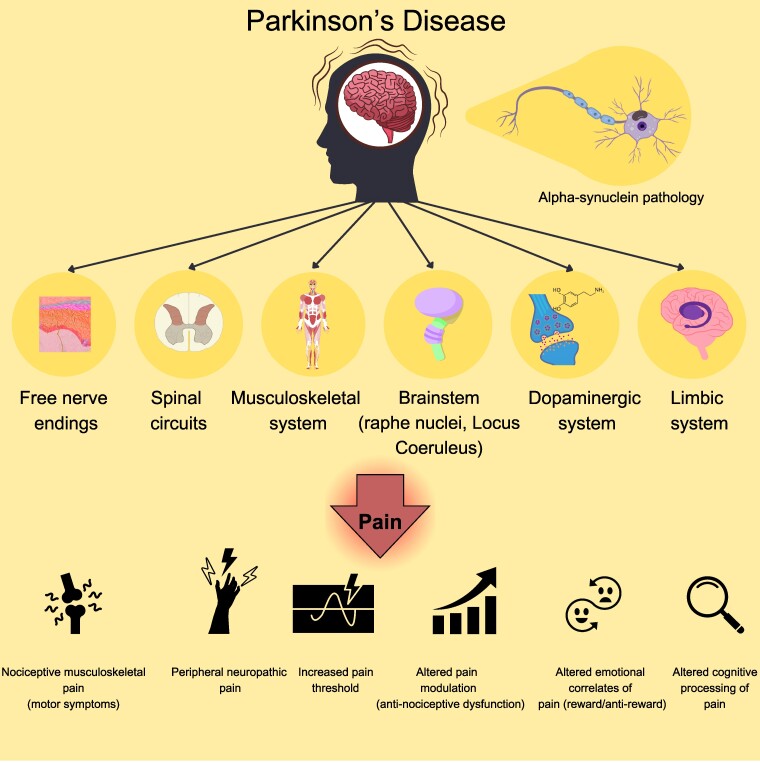

## Introduction

Parkinson's disease (PD) is the most common neurodegenerative disease after Alzheimer's disease in the population over 55 years of age.^1^

Motor symptoms are the ‘tip of the iceberg’ of a heterogeneous clinical spectrum, in which non-motor symptoms often occur before the onset of the motor phenotype. Thus, a precise definition and diagnosis of non-motor symptoms might foster the administration of potential disease-modifying therapies at the early stages of the disease.

Among non-motor symptoms, pain is frequently experienced by Parkinson's disease patients, requiring in depth investigation to clarify the downstream anatomical and pathophysiological mechanisms. Pain is defined by the International Association for the Study of Pain as: ‘an unpleasant sensory and emotional experience associated with—or resembling that associated with—actual or potential tissue damage’.^2^ In Parkinson's disease, pain can be present both in chronic and fluctuating forms,^3,4^ preceding the onset of motor phenotype in about 5% of patients affected by early Parkinson's disease. The burden of pain in Parkinson's disease might be, however, underestimated, since the main part of clinical studies have not precisely defined the unpleasant, generalized sensation, which is not experienced as epicritic or localized pain. Chronic pain, in fact, implies a biopsychosocial complexity of the unpleasant experience with a downstream complex anatomical network responsible of pain perception, modulation and processing. Interestingly, all the anatomical areas involved in this pain network can be affected by synucleinopathy, suggesting that pathophysiology of pain in Parkinson's disease encompasses a ‘pain spectrum’, involving different anatomical and neurochemical pathways.

In this article, we will first summarize the clinical burden of pain in Parkinson's disease, highlighting the limits of the current pain classification systems, which could benefit of a detailed anatomical definition. Thereafter, we will focus on various anatomical areas recruited in pain perception, modulation and processing, highlighting the potential consequences of their degeneration in course of Parkinson's disease. This review provides the first anatomically driven comprehension of pain in Parkinson's disease, aiming at fostering new insights for clinical diagnosis and therapeutic interventions.

## Clinical burden of pain in Parkinson's disease

The prevalence of pain in Parkinson's disease, ranges from 24% up to 85% of the Parkinson's disease population.^5-8^ It is a continuous pain in at least two-thirds of cases, fluctuating in about 30% of cases, and it invariably produces a dramatic impairment in the quality of life.^9,10^

In Parkinson's disease, the onset of pain may precede the development of motor symptoms by several years.^11^ In 5% of people with Parkinson's disease, pain represents the first clinical symptom. While the prevalence of pain in Parkinson's disease increases along with disease duration and in the advanced stages of the disease.^12^ Predictive factors in pain development are female gender, sleep disturbances, depression,^13^ dyskinesias, postural abnormalities and motor complications.^14,15^ Moreover, several non-neurologic comorbidities contribute to worsen pain in parkinsonian patients, including diabetes mellitus, osteoporosis, rheumatic diseases, and arthritis.^14^

Chronic pain in Parkinson's disease is heterogeneous for its quality and placement, which witnesses for a complex and multiple etiology.^16^ Ford's classification represents the most employed clinical classification of pain in Parkinson's disease, and it classifies pain mainly according to the relation with motor and non-motor symptoms and to its localization, distinguishing musculoskeletal pain, radicular/neuropathic pain, dystonia-related pain, akathisia discomfort and central pain.^17^ Moreover, Ford's classification also includes: the ‘coat hanger’ pain, related to orthostatic hypotension, the abdominal pain caused by constipation, and the pain associated with restless legs syndrome. However, Ford's classification neglects a precise mechanistic approach and a detailed description of the anatomical pathways underlying pain in pwPD. This gap produces an incomplete and unclear interpretation of the pathophysiological mechanisms related to each pain type. Particularly, the anatomical explanation of Parkinson's disease-related ‘central pain’, proposed by Ford, is almost undefined, encompassing under the definition of ‘medial spinoreticulothalamic pathway’ different structures of the spinal cord, the brain stem or the telencephalon, which have distinct neurophysiological roles in pain perception and modulation, and which employ distinct neurotransmitters systems.

Different scales have been employed to score and diagnose pain in pwPD, assessing different pain dimensions^18-21^ or specifically focusing on neuropathic pain.^22,23^ However, the main part of them lacks a specific validation in Parkinson's disease cohorts, reaching only a recommendation with caution for pain assessment in Parkinson's disease.^24^ The King's Parkinson's disease pain scale (KPPS) is the first validated scale for assessing pain intensity in Parkinson's disease, but it is only suggested for the syndromic classification of pain. It is mostly based on Ford's pain sub-types, although it introduces also additional pain categories, always related to specific clinical symptoms of pwPD: pain related to motor fluctuations, nocturnal pain and visceral pain.^25^

A taxonomy of pain in Parkinson's disease, based on aetiology, has been proposed by Wasner and Deuschl,^26^ who first differentiated nociceptive pain from neuropathic pain in Parkinson's disease. Based on this preposition, a comprehensive classification and scoring system for pain in Parkinson's disease has been provided by the Parkinson's disease pain classification system (PD-PCS), which starts from the differentiation between Parkinson's disease-related and Parkinson's disease-unrelated pain and then distinguishes three different pain types on the basis of pathophysiological mechanisms.^27^ Pain was considered as Parkinson's disease-related when occurring along with the first motor symptoms, when it is aggravated during the OFF stages or occurs in the context of dyskinesias or dystonia and when it is consistently improved by dopaminergic treatment.^17,28,29^ Conversely, pain is considered unrelated to Parkinson's disease, when it is neither started, nor aggravated by the occurrence of Parkinson's disease or its motor complications. According to this view, Parkinson's disease-related pain was classified through a mechanistic approach into neuropathic, nociceptive and nociplastic.

Neuropathic pain is distinguished in peripheral or central and encompasses all the painful conditions in which a damage of the pain sensory system occurs.^30^ Clinically it is deemed present when neuropathic pain questionnaire is scored positive.^22^ Anatomically, peripheral neuropathic pain in Parkinson's disease has been referred to those conditions in which, due to motor or postural abnormalities, spinal roots or peripheral nerves can be compressed, with the occurrence of pain located in the coherent innervation territories. While, in central neuropathic pain, central anatomical structures of the pain sensory system can be compromised, including basal ganglia-thalamocortical circuits or diencephalo-spinal pathways.^30^ This latter is typically described as diffuse pain, accompanied by the sensation of aching, burning or tingling. It can be closely related to motor symptoms, but it can also occur independently from them. Interestingly, the clinical features of Parkinson's disease-related neuropathic pain differ from the ‘classic’ central neuropathic pain, since in ‘classic’ central neuropathic pain deficits in pain and temperature sensation occur in the regions anatomically coherent with the central lesion, whereas these deficits are not always detectable in pwPD.^31,32^

Nociceptive pain arises from an actual or threatened damage to non-neuronal tissue, due to the activation of peripheral nociceptors. In pwPD, this has been referred to musculoskeletal pain related to fluctuations in the motor status, such as wearing-off pain, dystonia-related pain and dystonic spasms as well as to hanger headache and neck pain due to orthostatic hypotension. Differently from central neuropathic pain, nociceptive pain is always localized and always related to the motor or autonomic symptoms. Musculoskeletal pain is prevalently located at the upper extremities, in paravertebral region and in the neck.^17^ Consistently among different clinical studies, it is the most prevalent form of pain associated with Parkinson's disease^33^ and it is frequently responsive to parkinsonian medications. Pain associated with autonomic failure (also known as coat-hanger pain) is closely related to orthostatic hypotension and typically located in the neck and at the occipito-cervical region.^34,35^ Visceral pain due to constipation has been classified as visceral nociceptive pain.^36,37^

Finally, nociplastic pain is present when pain is neither neuropathic, nor nociceptive, comprising all the conditions in which the nociceptive system is overactive, without any evidence of somatosensory system lesion or peripheral nociceptive activation.^38,39^ In this condition have been included all the painful and discomfort syndromes related to dopaminergic fluctuations, when non-motor symptoms dominate the clinical picture and the patient can face—together with pain—sweating, psycho-motor agitation, inner restlessness or wandering. Typically, this kind of pain is mostly not localized and migrating or deeply located in the abdomen or in the face. Pain related to restless legs syndrome and akathisia discomfort have been assigned to this group. Patients with Parkinson's disease nociplastic pain have been classified as having dopaminergic agonists withdrawal syndrome or Dopamine (DA) dysregulation syndrome and a strong association with neuropsychiatric symptoms.^27^

The main limit of the PD-PCS consists of the prominent causative role attributed to motor symptoms in determining neuropathic pain in Parkinson's disease, although not all the studies converge on a direct correlation between motor impairment and pain development or severity.^40,41^ In fact, total PD-PCS score did not correlate with the MDS-UPDRS motor scores in the study by Mylius and coworkers,^27^ while in a consistent percentage of cases pain precedes of years the onset of motor symptoms,^42-44^ supporting a multifaceted pain aetiology in pwPD. Although reporting a comprehensive classification of pain in Parkinson's disease, based on pathophysiology, PD-PCS lacks in providing detailed neuroanatomical correlates for different types of central neuropathic and nociplastic pain, in which—apart from DA—various neuronal pathways of the somatosensory and limbic systems are involved.

Table 1 summarizes the classifications of pain in Parkinson's disease according to Ford^17^ and KPPS.^25^ Table 2 summarizes the PD-PCS.^27^

**Table 1:** Clinical classification of pain in Parkinson's disease according to Ford's Classification^17,25^

Pain subtypes	Prevalence	Clinical features	Comorbidities	Stage of the disease
Musculo-skeletal pain	40–75%	Joint pain, lower back pain and spinal paravertebral pain	Wearing-off, motor fluctuations, dyskinesias Exacerbated by: bone mineralization disorders and arthrosis or arthritis	Increased prevalence in the advanced stages of the disease and with increased MDS-UPDRS score and motor fluctuations
Radicular neuropathic pain	5–20%	Numbness and weakness in a nerve root territory	Truncal deformities: Pisa syndrome, kyphoscoliosis, camptocormia. Cervical/lumbar radiculopathies	Prevalence increases during the disease course
Polyneuropathic pain	37.8–55%	Dysesthesia, hyperalgesia and burning syndrome involving the lower extremities. Neurophysiological evidence of peripheral neuropathy	Deficit of vitamin B12 or folic acid. Increased prevalence in patients with intra-duodenal administration of DuoDopa gels	Increased prevalence at the advanced stages of the disease, but present also in PD at early stages
*‘Central pain’*	22%	Diffuse pain, without a precise localization. General sensation of aching, burning and tingling. General sensation of discomfort. Bizarre in quality. Not confined to root or nerve territories.	Non-motor symptoms, cognitive and behavioural symptoms	It can occur at different stages of the disease. Prevalent in case of dopamine withdrawal
Dystonic Pain	8–50%	Painful foot dystonia with a combination of toe inversion, eversion, plantar and dorsal flexion	Dyskinesias and dystonia Typically occurring in the morning after the first levodopa administration	Increased prevalence in the advanced stages and with the occurrence of Levo-Dopa-induced dyskinesias or Dystonia
Orofacial pain	4–24%	Pain when chewing, nocturnal teeth grinding and burning mouth syndrome	Abnormalities of the temporo-mandibular joint. Peripheral neuropathy	Increased prevalence at the advanced stages of the disease with the occurrence of orofacial dyskinesias
Coat-hanger pain	Up to 24%	Acute pain, typically located at the neck and at the occipito-cervical region	Orthostatic hypotension	It can occur at each stage of the disease. Typically associated to Levo-Dopa therapy
Visceral pain	Up to 24%	Acute and chronic pain localized at the abdominal region	Constipation	It can occur at the early stages of the disease also before motor symptoms. It can be associated with Levo-Dopa treatment

**Table 2:** Parkinson's disease pain classification system—^27^

Parkinson's disease-unrelated pain	Parkinson's disease-related pain	
Pain neither started or aggravated by Parkinson's disease and/or by motor symptoms and their complications	Pain started or aggravated by Parkinson's disease and/or by motor symptoms and their complications	
	Nociceptive	Due to discharge of peripheral nociceptors (musculoskeletal pain)
	Neuropathic	Due to lesions at the peripheral or central pain sensory system (peripheral nerves compression, compromission of basal ganglia-thalamo-cortical system)
	Nociplastic	Due to overactivity of the pain processing system (dopaminergic fluctuations associated with non-motor cognitive and behavioural symptoms)

## Anatomical correlates of pain in Parkinson's disease

### General concepts of pain processing in relation with Parkinson's disease

Noxious stimuli are perceived by nociceptors in first-order neurons by activating specialized ion channels that generate action potentials. Pain signals are propagated by unmyelinated C and myelinated A-*delta* fibres to second-order neurons in the spinal cord, where they are modulated by interneurons and local immune cells, and inhibited by descending pathways, originating from the rostral ventromedial medulla (RVM), the dorsolateral ponto-mesencephalic tegmentum and the periaqueductal grey (PAG). The descending pathways inhibit pain by releasing monoamines (especially norepinephrine [NE] and serotonin) and endogenous opioid neuropeptides into the dorsal horn.^45^ Then, pain signals are projected from the spinal cord to supraspinal regions by the lateral and medial pain systems. The lateral pain system is composed of the lateral spinothalamic tract, which projects toward the cortical sensory areas through the ventral thalamus [VPL and ventro-posteromedial nucleus (VPM) nuclei]. The lateral system is primarily involved in processing sensory discrimination, localization and pain intensity, comprised under the umbrella of epicritic pain. In contrast, the medial pain system processes the motivational-affective and cognitive-evaluative aspects of pain (e.g. unpleasantness, suffering). It projects through the ventral spino-thalamic tract to the medial and the anterior thalamic nuclei towards which it reaches the anterior cingulate cortex (ACC), the orbito-frontal cortex, the amygdala and the other structures of the limbic system. Abnormal pain processing in Parkinson's disease has been related to increased sensitivity of both lateral and medial pain pathways.^16^ Moreover, a maladaptive neuro-immune crosstalk in the context of gut dysbiosis has been recently proposed as contributor of pain hypersensitivity with the occurrence of neuroinflammation at different sites of the lateral and medial pain pathways.^46^

Neuropathologically, a-syn pathology damages different anatomical regions throughout the CNS. Synaptic failure in the striatum might be one of the earliest pathological events related to synucleinopathy,^47-49^ leading to impaired release of DA in the dorsal striatum and neuronal death in the SNpc.^47,49,50^ Besides the nigrostriatal pathway, degeneration in different anatomical sites determines a progressively worsening and multifaceted clinical picture. Therefore, inquiring the nature of pain in Parkinson's disease, we should consider a complex and multifactorial origin that involves the loss of DA in the nigrostriatal pathway, but that can be potentially associated to degeneration of other dopaminergic areas, as well as other crucial anatomical sites for pain control, such as the brainstem,^51^ the small peripheral nerve fibres and the spinal cord.^52^ Furthermore, given the impact of chronic pain in driving emotional responses, brain regions, involved in emotional processing or motivation-reward behaviours must be considered, including: NAc, amygdala and habenular complex.^53^

### The controversial role of small fibers neuropathy

Peripheral neuropathic pain in Parkinson's disease has been correlated to compressive radiculopathies, due to the occurrence of postural and vertebral abnormalities. A clear correlation between small fibres neuropathy and pain is not reported in Parkinson's disease. However, a high prevalence of small fibres alterations was observed in Parkinson's disease population, based on combined neurophysiological and pathological assessments.^54^ Non-length-dependent small fibre degeneration (SFD) appears to be among the earliest manifestations of Parkinson's disease, occurring at the prodromal phase.^55,56^ Reduced density of small nervous fibres in the skin correlates with thermo-nociceptive abnormalities and with impaired sudomotor function and lower sympathetic skin response, suggesting the involvement of both sensory and autonomic small fibres.^54,57,58^ The SFD does not appear to stem as a secondary effect of levodopa therapy, rather, it likely originates from neurodegenerative processes,^54,57,59^ since in the majority of cases precedes the onset of motor symptoms and the starting of parkinsonian medications. Accordantly, newly diagnosed Parkinson's disease patients untreated with levodopa, already feature some extent of peripheral nerve dysfunction detected in small corneal fibres.^55,60^ The presence of a-syn aggregates has been reported in small cutaneous fibres of Parkinson's disease patients, using Proximity ligation assay (PLA) and correlates with SFD and with motor and non-motor scores.^59^ Moreover, several studies have detected a-syn pathology in the small fibres of the skin and have reported its potential application as biomarker for the diagnosis of synucleinopathies.^61^ Small fibres reactive for phosphorylated alpha-synuclein (p-a-syn) have been detected in the skin of patients affected by Parkinson's disease and by pure autonomic failure.^62-66^ While a-syn deposition has been reported in Schwann cells and has demonstrated high sensitivity and specificity in differentiating Parkinson's disease from Multiple System Atrophy.^67^ Furthermore, a-syn aggregation assays, including RT-QuIC, have demonstrated a-syn seeding activity in the skin of Parkinson's disease patients^68^ and in the skin of patients affected by REM behavioural disorder, a prodromal clinical condition for synucleinopathies.^69,70^

The hypothesis that peripheral neuropathy leads to the development of neuropathic pain in pwPD is suggested by several studies conducted in small cohorts of patients,^71-73^ but remains controversial. Occurrence of peripheral neuropathy, with loss of small nervous fibres, reduces pain threshold and induces nerve ectopic activity^74-77^ through altered activity of voltage ionic channels and hyperexpression of pain receptorial proteins.^78^ Moreover, peripheral neuropathy induces central sensitization of second-order post-synaptic neurons in the spinal cord. Molecular changes in these neurons induce hyperexcitability enabling low-threshold mechanosensitive A-*beta* and A-*delta* fibres, overactivating pain transmission, with the result of hyperalgesia and mechanical allodynia.^79,80^ Multiple studies have suggested that pwPD have lower pain threshold when compared to general population. Significant lowering in both mechanical and thermal pain thresholds have been reported in pwPD on respect to controls subjects,^81-85^ together with hyperalgesia and mechanical allodynia.^86,87^ However, reduced pain thresholds resulted aggravated by the OFF state and variably responsive to dopaminergic therapy,^81,88^ supporting the link between DA loss and pain sensitization,^81^ rather than a reduced pain threshold due to peripheral neuropathy. On the other hand, chronic hypoalgesia following a period of acute hyperalgesia has also been observed in pwPD,^89^ while Nolano and co-workers^57^ reported chronic hypoalgesia and elevated mechanical and thermal pain thresholds, in correlation with peripheral small fibres neuropathy detected in skin biopsies. In addition, the perception of affective touch was affected in a cohort of 27 pwPD and positively correlated with the reduced density of cutaneous C-fibers.^90^ Synucleinopathy in peripheral nerve fibres is a widely accepted neuropathological feature of Parkinson's disease and prevalently involves long, unmyelinated small fibres.^91^ Moreover, altered metabolism of vitamin B12, further contribute to small fibres loss in pwPD at the advanced stages.^92^ How peripheral neuropathy contributes to chronic neuropathic pain in Parkinson's disease is controversial. It is possible that small fibres degeneration precedes motor symptoms, determining an altered pain threshold, exacerbated by the progressive dopaminergic loss. Conversely, synucleinopathy could lead to the development of hypoalgesia and protopathic hypoesthesia. Further studies correlating clinical evidence of neuropathic pain with neuropathological and neurophysiological data in large Parkinson's disease cohorts are mandatory to clarify this aspect.

### Spinal circuits: nociceptive neurons and modulatory interneurons

The posterior laminae of the spinal cord—corresponding to the Lissauer tract (lamina I) and the substantia gelatinosa (lamina II and dorsal lamina III)—represent the first centre for pain transmission and modulation, and are particularly involved in establishing pain threshold, in the modulation of the intensity of pain perception, and in intertwining pain perception with other sensory modalities. For this reason, the dorsal laminae of the spinal cord are characterized by a complex network of interneurons and are gathered by different neurotransmitters pathways arising from the diencephalon and the brainstem.

**Figure 1:**
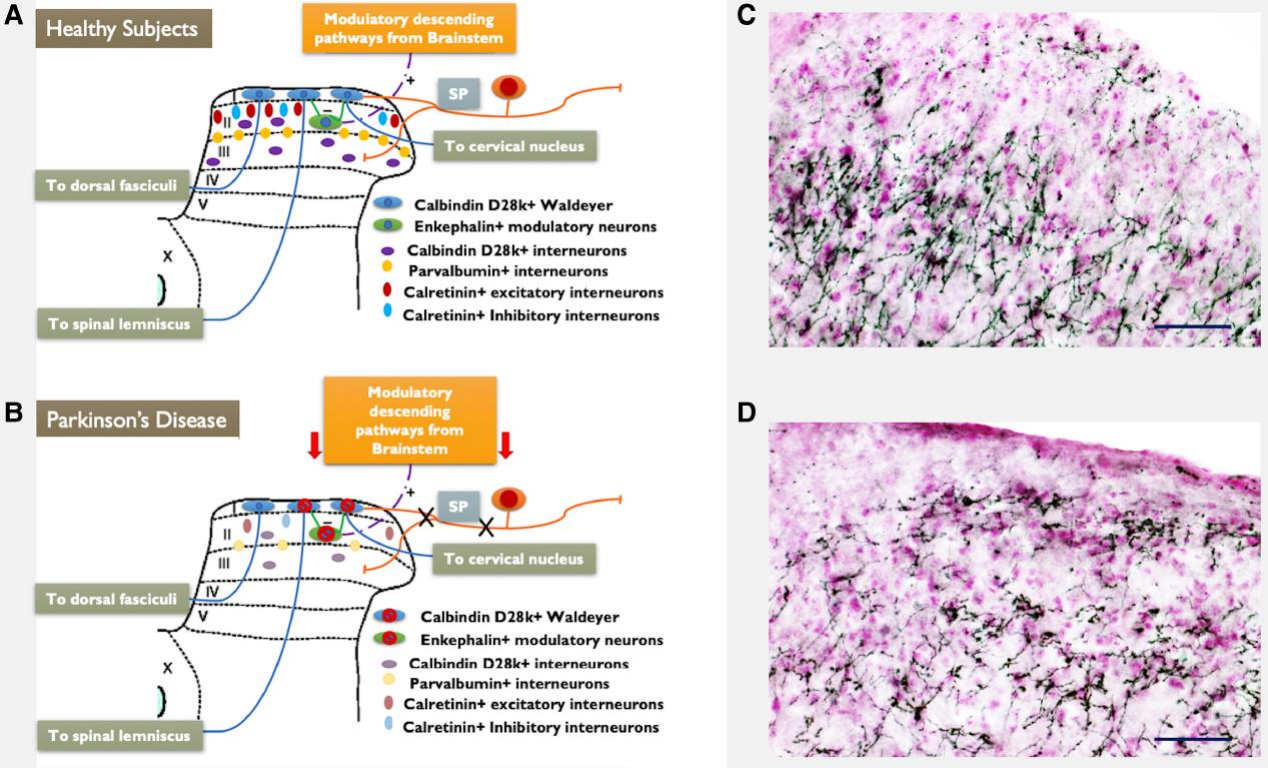
**Spinal circuitry of pain in Parkinson's disease.** Schematic representation of the altered anatomical circuits occurring in the posterior laminae of the spinal cord in course of Parkinson's disease. The degeneration of different neuronal populations involved in the spinal processing of pain is reported. Neurochemical and topographical organization of the pain projecting neurons and of the interneuronal populations of the posterior laminae of the spinal cord is schematically represented in **A**. Whereas in **B**, possible patterns of neurodegeneration of the same neurons in course of Parkinson's disease is reported. **C** and **D** report immunohistochemistry for Tyrosine Hydroxylase (TH—Millipore anti-TH AB1542. 1:10 000–4°C for 72 h) in the posterior laminae of the spinal cord in healthy mice **C** and in experimental parkinsonism obtained by intraperitoneal administration of MPTP **D**. Black stained structures are TH positive fibres. Cell nuclei are in light purple, counterstained by neutral red. Wide-spreading TH positive fibres are detectable in laminae I, II and III, where they result drastically reduced after exposure to MPTP, supporting the loss of NA and dopamine modulation in the spinal circuits as a possible pathogenetic mechanism of pain in Parkinson's disease. Linear bar 50 µm.

In our recent study we found that the posterior laminae of the spinal cord are affected by a-syn pathology in 1 methyl-4 phenyl tetrahydro-pyridine (MPTP) models of parkinsonism and that different inter-neuronal subtypes in the posterior laminae were lost^93^ (Fig. 1A and B). In particular, after chronic exposure to MPTP we have detected roughly 40% neuronal loss in the *substantia gelatinosa* (lamina II and dorsal lamina III). Accordingly, previous autopsic studies have shown neuropathological involvement of spinal laminae I, II and III in Parkinson's disease, where nociceptive neurons and interneurons show a-syn pathology.^94^ This is consistent with alpha-synuclein pathology affecting nociceptive amyelinic fibres early in Parkinson's disease,^94^ which were reported to accumulate alpha-synuclein in both humans and rodents.^95,96^

Among interneurons, calretinin interneurons are the largest population in laminae I and II of the spinal cord.^97,98^ Physiologically, 75% of calretinin positive interneurons are considered excitatory, whereas 25% of these neurons represent a sub-population known as ‘islet cells’ and expressing SP.^99,100^ Activation of excitatory calretinin positive cells is reported to cause a reduction of von Frey threshold and facilitation of transmission of mechanical stimuli,^101,102^ such a condition is reproduced in MPTP models of parkinsonism.^103^ On the other hand, loss of islet cells (inhibitory calretinin interneurons placed in the outer layer of lamina II) might facilitate transmission of pain inputs form the periphery, by disinhibition of glutamatergic vertical cells and consequent over-activation of pain projecting neurons of lamina I. Calbindin D28K positive neurons are mainly present in lamina I and in the outer layer of lamina II.^104^ Calbindin D28K positive interneurons of laminae I, II and III are morphologically and physiologically heterogeneous. Some studies have shown that lamina I owns mainly projecting neurons sensing pain and temperature, which send their axons to the VPM of the thalamus.^105,106^ Neurons within lamina II and dorsal lamina III, instead, are mainly considered as interneurons, involved in the modulation of pain and somatosensory inputs, through both excitatory and inhibitory activity.^107^ Parvalbumin positive neurons are inhibitory GABAergic or glycinergic interneurons, the cell bodies are placed within lamina III and their axons move dorsally to internal-ventral layer of lamina II, taking part to a complex network of interneurons which modulates the vertical glutamatergic cells of the outer layer of lamina II. These neurons are supposed to activate, projecting sensory neurons of lamina I. Loss of parvalbumin inhibitory interneurons may disinhibit vertical glutamatergic cells thus eliciting pain transmission. Moreover, parvalbumin interneurons are also activated by myelinated Aβ fibres transmitting mechanical stimuli. Their activation is needed to prevent the engagement of nociceptive network during light touch. The loss of these interneurons may explain the neurophysiological and behavioural features of mechanical allodynia reported in MPTP treated animals^103^ and might also explain mechanical hypersensitivity reported in Parkinson's disease patients.^108,109^ Met-Enkephalin neurons are crucial for the modulation of pain transmission. They receive afferent nociceptive fibres from the periphery, and they are activated by descending serotoninergic and noradrenergic pathways from the brainstem. Activation of Met-Enk neurons leads the inhibition of projecting spino-thalamic neurons of lamina I as well the axo-axonic inhibition of fibres A-delta and C coming from the periphery and entering the spinal cord at laminae I and II. Disruption of Met-Enk network contributes to disinhibition of pain transmission and might represent a further mechanism underlying the development of chronic pain in experimental parkinsonism and in Parkinson's disease.

The potential role in Parkinson's disease-related neuropathic pain of a degenerative process at the spinal cord is almost based on neuropathology and pre-clinical evidence. Clinical and post-mortem studies correlating degeneration of the spinal cord with the incidence of neuropathic pain in Parkinson's disease population are currently unavailable. However, the overall anatomical picture suggests that a-syn pathology in the posterior laminae of the spinal cord is likely to create a scenario in which intra-spinal pain modulation is disrupted, contributing to hyperalgesia, reduced pain threshold and mechanical allodynia in pwPD.^108,109^

### Locus coeruleus

According to Parkinson's disease staging proposed by Braak *et al*.,^110^ locus coeruleus (LC), alongside with caudal raphe nuclei and magnocellular reticular formation is affected as early as in stage 2. In particular, SNc and LC neurons—retaining high concentrations on neuromelanin (NM)—appear to be the main two populations targeted in early and mid-stage neurodegenerative pathology.^111^ NM in fact, plays a recognized neurodegenerative role in Parkinson's disease.^112,113^

The mechanism subtending increased vulnerability of catecholaminergic neurons may depend on the process of auto-oxidation of catecholamines,^114^ but may also lie in the high levels of MHC-I in NM-containing organelles of LC neurons. In fact, the expression of MHC-I appears to be exclusive of specific neurons affected by Parkinson's disease pathology.^115^ As a consequence, CD8 + cells were observed in proximity of NM-rich neurons expressing MHC-I in SN and LC, harbouring cytotoxic effects.^115^ In the same neurons, a neuroinflammatory state is chronically perpetuated by a devastating microglial activation and by release of insoluble NM granules from dying neurons into the extracellular space.^114,116,117^ In cell cultures, NM appears to be a major activator of innate immunity as well, being a positive chemotactic driver, an activator of pro-inflammatory transcription factor kappaB (NF-kB) with consequent up-regulation of tumour necrosis factor alpha, interleukin-1 and nitric oxide.^118^

Zarow *et al*.^119^ have demonstrated that the LC is the site of greatest subcortical neuronal degeneration, in both Parkinson's disease and Alzheimer's disease, with the average LC cell loss being of 67.9% in Alzheimer's disease (variably distributed) and 83.2% in Parkinson's disease (uniformly severe across all cases). Lewy bodies in LC have been consistently observed in Parkinson's disease patients.^120^ Furthermore, loss of LC–Noradrenaline (NA) transmission in Parkinson's disease patients reverberates on dopaminergic populations and leads to both increased vulnerability of DA neurons and impaired recovery of nigrostriatal DA pathway.^121^

Neuronal degeneration in the LC correlates with important non-motor symptoms of Parkinson's disease, which manifest in prodromal stages of disease.^122^ It is estimated, in fact, that the onset of LC degeneration occurs more than 10 years before clinical diagnosis of Parkinson's disease.^110^ Among non-motor symptoms, chronic pain might also be related to the loss of NA projections arising from the LC. Accordantly, different neuroanatomical regions involved in the pain network are targeted by LC projections^121,123-125^ and NA loss is likely involved in all types of Parkinson's disease-related pain, including neuropathic, nociceptive and nociplastic.^27^

The LC is a critical nucleus for pain modulation through descending analgesic pathways.^126^ LC activation in response to pain, results in the release of NA into the laminae I, II and III of the spinal cord (dorsal horn—DHSC), leading to inhibition of nociceptive neurons, through the activation of enkephalinergic inhibitory interneurons of lamina II.^51,127,128^

In chronic pain, an increased release of NA to the DHSC has been reported, which notably exerts negative feedback on pain perception.^129^ In accordance, electrical stimulation of the LC can effectively suppress neuropathic pain^130^ and pharmacological approaches inhibiting the reuptake of NA are widely used to treat neuropathic pain syndromes. In Parkinson's disease, the negative feedback exerted by NA on the DHSC is likely to be ineffective, due to the depletion of most noradrenergic neurons in the LC. Nevertheless, descending NA projections to the DHSC are involved in a wide range of neuroprotective mechanisms such as significant inhibitory effects on microglial activation and neuroinflammation,^131^ prevention of oxidative stress and promotion of cell survival pathways.^132,133^ Inhibition of neuroinflammation and microglial activation might represent a further mechanism for LC antinociceptive activity, due to the contribution of inflammation in the sensitization of nociceptive neurons.^134,135^ Conversely, in pwPD noradrenergic fibres are directly involved by neuroinflammation and they may paradoxically act as chemotactic drivers of microglial activation and cytokines production through the release of NM granules.^114,116-118^

Loss of LC integrity is reported to facilitate the onset and the worsening of L-DOPA-induced dyskinesias,^121^ by abolishing the modulatory effect of NA on D1 receptors.^121,136,137^ In accordance, loss of NA due to neurodegeneration of the LC might be also involved in the development of musculoskeletal nociceptive pain, related to dyskinesias.^16,17^

A complex interaction among brainstem pathways and their receptors modulates both inhibition and facilitation of pain processing.^138^ LC neurodegeneration with loss of NE projections is likely to affect pain sensitivity and tolerance through different mechanisms, including the modulation of DAergic transmission in the NAc and the direct NE transmission to the thalamus and the striatum.

The NA system exerts neuroprotective effect on ventral tegmental area (VTA) DA neurons,^121^ while LC projections to the VTA have a key role in homeostatic plasticity of DA neurons,^139^ fostering DA neurons activity through α1- and β3-adrenergic receptors (ARs).^140,141^ Moreover, prefrontal cortical NE controls DA transmission in the NAc, spurring it through activation of prefrontal cortex α1-ARs^142-144^ Therefore, LC degeneration, reduces NAc DA transmission, due to an altered VTA stimulation (altering DA transmission to the forebrain) as well as to the loss of NA in mPFC, which further impairs DA transmission in the NAc. Prefrontal NA transmission is critical for attribution of motivational salience (modulated by DA in the NAc) to both rewarding and aversive stimuli,^142,145^ indicating that the catecholamine prefrontal-accumbal system regulates affective states related to aversive stimuli and highlighting its potential role in nociplastic pain. Furthermore, a dramatic loss of NA neurons of the LC has been reported in Parkinson's disease patients with depression, though the use of Positron Emission Tomography (PET),^146^ while reduced binding rate to Norepinephrine transporters (NET) has been detected in Parkinson's disease patients with anxiety, in both the striatum and the thalamus.^147^ Notably, Parkinson's disease patients experiencing depression report significantly higher incidence of chronic pain and significantly lower pain threshold in comparison to those without depression.^87,148,149^ These data support a direct role of NA transmission in Parkinson's disease-related nociplastic pain.

### The multifaced role of dopamine and basal ganglia

Dopaminergic dysfunction is the prominent neuroanatomical and neurophysiological alteration in pwPD. Data surfacing from human and animal experimental studies indicate that all the DA neuronal pathways [VTA, SNpc and posterior hypothalamic area (PHA)] are extensively involved in pain perception and modulation. Accordantly, clinical and experimental evidence support the close relationship between DA and Parkinson's disease-related pain, with DA loss and dysregulation detrimentally contributing to either nociceptive, as neuropathic and nociplastic pain mechanisms.

Pain in Parkinson's disease was related to motor fluctuations.^8^ Musculoskeletal nociceptive pain, in fact, depends on the discharge of peripheral nociceptors in response to potential injuries at the musculoskeletal system, which occurs in association with Parkinson's disease motor symptoms. For instance, muscular rigidity and dyskinesias can induce pain at the joins and postural abnormalities can be themselves a direct source of discomfort or the responsible of radiculopathies. Accordantly, pwPD referring musculoskeletal pain have a higher UPDRS part III and a longer disease duration.^150^ Musculoskeletal nociceptive pain is variably experienced at different points of the dopaminergic treatment. It can occur during the wearing-off or in association with dyskinesias.^29^ Due to its direct relation with parkinsonian motor symptoms, nociceptive pain frequently responds to dopaminergic therapy, it is attenuated during the on-state, and it is inversely correlated to dyskinesias.^151^

Besides musculoskeletal nociceptive pain, central nociplastic mechanisms closely associate DA to Parkinson's disease-related chronic pain. Many studies have reported an increased pain sensitivity in pwPD in comparison to HS, and a recent meta-analysis supports a reduced pain threshold in Parkinson's disease across all pain modalities.^152^ Interestingly, there were no significant differences in pain threshold between patients with or without clinical evidence of Parkinson's disease-related pain,^88^ suggesting a general oversensitivity to pain in Parkinson's disease, which likely depends on dopaminergic dysfunction. Neurophysiological studies demonstrated that cortical pain processing was altered during OFF and unmedicated periods, whereas it became again normal in ON periods and under dopaminergic medication.^85^ Levodopa or direct DA agonists increase pain threshold and multimodal pain threshold is responsive to levodopa treatment and dosing.^81,153^ Moreover, after Deep Brain Stimulation (DBS), patients have shown a significative modification of pain thresholds towards normal values, further suggesting a central nociplastic mechanism involving the basal ganglia.^154^ Nociplastic mechanisms related to DA also affect the emotional and affective aspects of pain in pwPD. PET targeting D2 and D3 DA receptors has shown alterations in both discriminative and affective-motivational pain processing in pwPD^155,156^ and recently, a direct association has been reported between Parkinson's disease-related musculoskeletal pain and the severity of the dopaminergic deficiency in the caudate nucleus.^157^

Mesostriatal and mesolimbic dopaminergic pathways have different role in pain perception and therefore are determinant for different aspects of pain perception and modulation in pwPD.

Meso-striatal DA system plays a role in setting the ‘pain threshold’, through the activation of D2-like receptors (D3), which have been also involved in the perception of tonic pain.^158^ Indeed, electrophysiological studies have demonstrated that specific groups of neurons within the basal ganglia encode stimulus intensity and are preferentially activated by noxious stimuli.^23,73,159^ Moreover, the dorsal striatum is connected to the descending pain modulatory system and in particular to the RVM through the medullary dorsal reticular nucleus, by which it exerts a robust anti-nociceptive effect on the intra-spinal pain circuits.^160^ In line with this, meso-striatal DA depletion causes mechanical hypersensitivity and decreased pain threshold in animal studies,^161^ which are both responsive to dopaminergic agonists and apomorphine.^162-164^ Impairment of the meso-striatal DA system, therefore, may determine pain oversensitivity in pwPD, which mainly concerns the setting of pain threshold^165,166^ and is responsive to both DBS and dopaminergic therapy.

On the other hand, VTA and mesolimbic DA system respond heterogeneously to pain perception,^53^ with some DA neurons suppressed and some others physically excited by nociceptive stimuli, which support the potential role of the VTA in modulating behavioural responses to pain. It is likely that VTA concomitantly inhibits pain by increasing DA firing to the NAc and PFC or elicits pain by reducing the DA output to the same areas. In keeping with this, a loss of tonic DA release in the NAc is associated with decreased pain tolerance, whereas DA release in the shell-NAc leads to reward and pain relief.^167^ Multiple rodent studies support this view, showing that noxious stimuli affect DA release in the brain reward centres, including the shell-NAc and the ACC, where specific changes in gene expression (of genes commonly associated with depression and anhedonia) occur, in response to chronic pain^168^.

In classic studies, meso-cortico-limbic DA neurons were shown to form part of a pain-suppression system.^169,170^ This system, classically considered as the core of the brain reward system, processes the salience and valence not only of rewarding but also of aversive stimuli. Reward and pain suppression close relationship leads to consider them on a *hedonic continuum* with extreme negative and positive affect located at opposite ends and normal affect located in the middle, in a *reward/anti-reward view.*^171^ Advanced methods involving transgenic animals, opto- and chemogenetics, have better unveiled the separate meso-limbic dopaminergic networks exerting opposite effects on pain. One of these networks stems from the medial VTA to innervate the NAc shell and produces analgesia through D2Rs. In the parallel network, instead, the DA neurons of the lateral VTA project to the NAc core, where DA enhances pain by its inhibitory effect on the D2Rs.^172-174^ In line with this view, painful stimuli increase DA release in the NAc core, as suggested by both rodent and human studies, while suppressing DA neurotransmission in the NAc shell.^103,167,175-177^ NAc DA has a crucial role in both transition to neuropathic pain and in stress-induced analgesia.^178-180^ Moreover, DA correlates with the intensity of perceived pain,^176,177^ thus indicating that DA response in the NAc is also associated with the affective correlates of pain. DA transmission in the NAc is also modulated by prefrontal-cortical DA in an inhibitory way,^181,182^ being accumbal DA response inversely correlated to the meso-cortical DA response. It follows that an increase in cortical DA transmission caused by adverse stimuli, such as pain, can translate into a reduction in dopaminergic transmission in the NAc-shell, blunting positive emotional states able to dampen the painful experience.

Furthermore, the emotions processed in the limbic areas influence and mediate pain perception by the descending pathway arising from dorso-medial PFC, ACC or amygdala and projecting downwards to the PAG, which is the primary centre of the descending antinociceptive system.^183^ DA injection into the ACC attenuates pain-related behaviour.^184^ DA fibres projecting from the VTA to the a-granular insular cortex inhibit nociception by activating GABAergic interneurons which in turn inhibit insular neurons projecting to the amygdala and to the limbic sub-cortical structures involved in motivational and affective aspects of pain.^185,186^ Moreover, they might also attenuate the autonomic response to pain by the inhibition of the projections from the insula to the hypothalamus.

Dopaminergic projections from the hypothalamus to the spinal cord represent a further anatomical pathway for pain modulation which can be affected in Parkinson's disease. Hypothalamic dopaminergic neurons project from the PHA to the laminae I and II of the spinal cord towards the diencephalo-spinal pathway.^187,188^ Recent studies have also highlighted the anti-inflammatory role of DA, which acts on the DA receptors expressed by microglia and astrocytes, inhibiting the NLRP3 inflammasome activated by several noxae, including misfolded a-syn.^189,190^ Several studies have shown the central role of neuroinflammation around sensory neurons in pain sensitisation,^131,191^ thus highlighting that DA loss occurring in Parkinson's disease may elicit pain perception by dysregulation of neuroinflammatory response.

The role of DA in pain perception results multifaceted and different mechanisms may relate DA loss with Parkinson's disease-related pain. Motor symptoms and motor fluctuations, due to dopaminergic dysfunction in the motor striatum, correlate with musculoskeletal nociceptive pain. The lack of DA in dorso-striatal and nigrostriatal DA pathway leads to the pain oversensitivity and reduced pain threshold. On the other hand, DA loss in the mesolimbic and mesocortical pathways might determine a reduced tolerance to pain and a reduced recruitment of the descending anti-nociceptive systems. Chronic pain affecting pwPD may reduce and/or disrupt reward-associated behaviours, in the same way in which reward-decision models illustrate the opposite situation, where significantly pleasant stimuli may decrease the perception of pain.^192^ DA may act as reinforcement stimulus counteracting nociception acting on the shell NAc, but dysregulated and unbalanced dopaminergic output from the VTA can also enhance pain perception by acting of on the NAc core. Finally, dopaminergic dysfunction may affect descending anti-nociceptive systems and recent studies have highlighted the role of DA as inhibitor of neuroinflammatory pathways involved in pain sensitization at the level of the spinal cord.

### A glimpse on clinical trials validate multiple roles of dopamine

The heterogenous role of DA is further highlighted by double-blinded clinical trials with dopamine agonists. Indeed, in an exploratory double-blind clinical trial, the effect of rotigotine on Parkinson's disease-related pain was evaluated.^193^ Sixty patients with advanced and at least moderate Parkinson's disease-related chronic pain were randomized 1:1 to receive rotigotine or placebo, for 12 weeks. Change in pain severity was considered as primary outcome together with KPPS domains. A numerical, but not significant, improvement in pain was observed in favour of rotigotine and the proportion of responders was rotigotine: 60% versus placebo: 47%. Interestingly a ∼2-fold numerical improvement in KPPS domain ‘fluctuation-related pain’ was also observed with rotigotine versus placebo. On the other hand, an inconsistent effect on pain score was reported by the employment of oxycodone-naloxone in a wide cohort of Parkinson's disease patients with various degrees of disease severity (H&Y I-IV).^194^ Taken together these results support the view that DA treatment is consistently efficient on Parkinson's disease-related muscolo-skeletal pain associated to motor fluctuations, whereas the efficacy on other pain domains is not constantly present, supporting the multifaced role of different DA pathways in pain processing.

### Amygdala: emotional and cognitive processing of pain

The amygdala is a bilateral structure located in the medial temporal lobe. It is part of the limbic system, as endorsed from its visceral and emotional functions.^195^ In fact, the amygdala plays a pivotal role in attributing emotional significance and in coupling emotions with autonomic responses, as first postulated by Kluver and Bucy in 1939 who identified a ‘psychic blindness’ in animals affected by amygdala lesions.^196,197^ Anatomically the amygdala is composed of three major nuclear groups^198^: the deep or *basolateral group*, which contains the lateral nucleus, the basal nucleus, and the accessory basal nucleus; the *superficial or cortical-like group*, which contains the cortical nuclei and the nucleus of the lateral olfactory tract; the *centromedial group*, which contains the medial and central nuclei. To this canonical classification, other amygdaloid nuclei must be added, such as the anterior amygdaloid area, the amygdalo-hippocampal area, and the intercalated cells.^199^ In addition, a rostro-medial extension of the centromedian amygdala into an area known as extended amygdala has been proposed.^200^

Braak *et al*.^201^ were the first to attribute a particular relevance to the amygdala as involved in course of Parkinson's disease. They postulated an invariable involvement of the accessory cortical nucleus and the CeA in Parkinson's disease, consistent across individual cases. Temporarily, they allocate the presence of inclusion bodies in these nuclei as early as in Braak Parkinson's disease stage 4, together with ventral claustrum and the interstitial nucleus of stria terminalis.^110^ Further corroborating this evidence, post-mortem studies have shown that the amygdala and the olfactory bulb are the most vulnerable sites of single-location a-syn pathology,^202^ since they contain large amounts of a-syn even in physiological conditions.

Recently, two main Parkinson's disease subtypes have been proposed: a body first model and a brain first model.^203^ In the body first (bottom-up) subtype, the pathology is supposed to arise in the enteric or peripheral autonomic nervous system, later ascending to the CNS through the vagus nerve and the sympathetic trunks, while in the brain first subtype (top-down), a-syn pathology rises first in the CNS and later spread peripherally to the autonomic nervous system. The amygdala is proposed as the site of origin of a-syn inclusions in brain first Parkinson's disease patients.^204^ Accordantly, a longitudinal study conducted by Pieperhoff *et al.*^205^ using deformation-based morphometry showed a superimposition between patterns of brain atrophy in Parkinson's disease patients and the patterns of a-syn deposition typically observed in Braak stages 3–5, including the atrophy of the amygdala, which strictly correlates—in turn—with NMSS scores, including the items for anxiety and pain.^206^

Indeed, the role of the amygdala in generating the emotional-affective state might be crucial in pain overlapping with aversion, anxiety and fear.^207-209^ The amygdala plays both a facilitating and inhibitory role on pain modulation, based on a polymodal integration of environmental conditions and affective states. The major amygdala outputs involved in pain processing and modulation are the projections of the CeA to the ventro-lateral PAG (vlPAG)^210-212^ and to the dorsal raphe nuclei (DR)^213-215^ which modulate pain via the descending antinociceptive system. The relationship of these two brain-stem regions with the CeA is strongly modulated by emotional states and environmental circumstances, resulting in either analgesic or algesic effects.^209,216^ Moreover, CeA projects to the parabrachial nucleus (PBN)^217,218^ and to the solitary tract nucleus,^219,220^ coupling pain processing with downstream autonomic responses.

The dense GABAergic population of the CeA can be functionally divided into Central medial amygdala (CeM), lateral central amygdala (CeL) and capsular central amygdala (CeC),^209,221,222^ where the CeC is the designated sub-nucleus receiving direct inputs from the PBN via the spino-parabrachio-thalamic tract, thus referred to as ‘nociceptive amygdala’.^208^ CeC display both local projections to the CeL and CeM, and extra-amygdaloid projections to PBN, thalamus, hypothalamus, hippocampus and PAG.^209^ This area features both nociceptive specific and multireceptive neurons which can also respond to innocuous cues and integrate nociceptive signals with affective information coming from the lateral-basolateral pathways.^223^ The BLA, better described as lateral-basolateral (LA/BLA), is made up of pyramidal glutamatergic projecting neurons receiving polymodal sensory information (including nociceptive inputs) from midline posterior nuclei of the thalamus, insular cortex, sensory association cortices, as well as from the NAc and medial prefrontal cortex (mPFC).^207-209,224^ Through this circuits, sensory inputs receive emotional-affective correlates, and this highly processed information are transmitted to the CeA for further processing within the amygdala fear circuitry.^225^ Finally, the ITC represent a GABAergic interneuron population, containing neuropeptide S (NPS) and opioids as co-transmitters.^226-228^ These neurons receive excitatory inputs from the infralimbic and prelimbic cortices and from the LA/BLA and they are activated during the extinction of pain and negative emotional responses^229,230^ exerting a pivotal role in the extinction of anxiety and fear.

The interactions between the BLA and the PFC are reciprocal and selectively involve cortico-amygdaloid neurons.^231,232^ The neurochemical integration occurring between BLA and mPFC is crucial for pain modulation. The nature of BLA projections to the infralimbic and prelimbic mPFC is glutamatergic, but the overall effect of these inputs is inherently inhibitory.^231-233^ While direct synapses with pyramidal cells interest fewer BLA axons, the strongest connections preferentially interest parvalbumin and somatostatin expressing interneurons, which act selectively on pyramidal neurons through GABAergic connections mediated by non-N-methyl-D-aspartate receptors and mGluR1.^223,231,234^ These monosynaptic connections interesting two parallel interneuron populations lie the foundations for parallel pathways of robust glutamate-driven feedforward inhibition to the mPFC, during pain stimulation.^231^ In fact, BLA hyperactivity leads to amygdala-driven mPFC deactivation, as demonstrated by decreased activity of output neurons in the infralimbic and prelimbic mPFC in acute and chronic pain models.^223,235,236^ In turn, mPFC deactivation reverberates on control of amygdala processing, reducing the firing of the BLA projections.^207^ This interaction between BLA and mPFC is crucial also for cognitive correlates of pain and chronic neuropathic pain has been in fact related to development of cognitive impairment.^235^ Furthermore, corticolimbic reverberating loops anticipate pain chronicization and interestingly, degeneration in the mPFC-BLA-NAc circuit together with a reduced volume of the amygdala have been associated with an increased risk of persistent pain.^237^

Neuropeptides are crucial for emotional and pain responses within amygdaloid circuitries. Calcitonin gene-related peptides (CGRP) play a pivotal role in nociceptive afferents from the PBN and mediates the excitatory tone of CeA neurons. Corticotropin releasing factor (CRF) and somatostatin (SOM) serve as long-range projections from the CeA to the target areas, including the DR and PAG. CRF has a crucial function in coupling pain with fear emotional conditioning, having excitatory function on both CeA and BLA. Over-population of CRF-expressing neurons in the CeA appears to be an important factor contributing to chronic pain development.^216,226^ NPS plays a gatekeeping role to the amygdala output, being associated with inhibitory ITC neurons.^227,228^ Whereas complex and intertwined functions are played by the opioid peptides (b-endorphin, enkephalin, dynorphin) acting on mu, delta and kappa receptors (MOR, DOR, KOR). Enkephalinergic (Enk) interneurons are the richest population of CeA interneurons, and they have a modulatory role on amygdala projections including nociceptive circuits. Enk neurons activation is thought to activate non-serotoninergic neurons in DR and vlPAG, acting on the descending pain inhibitory system^211,238^ and exerting antinociceptive and extinction effects like the Enk neurons within the spinal cord lamina II.

Overall, the function of the amygdala in pain processing and the differential role attributed to CeA, BLA and ITC, suggest that disruption of the amygdaloid circuits in Parkinson's disease can result either in abnormal emotional or cognitive processing of pain, as in deregulation on the descending anti-nociceptive pathways. Hypoconnectivity of all subregions of the amygdala was associated with pain in a recent functional MRI study.^239^ Moreover, functional brain connectivity comparing pwPD with DA-responsive or non-responsive pain and pwPD without clinical evidence of pain, revealed an altered connectivity of the amygdala with thalamus and putamen in patients with DA unresponsive pain on respect to the other groups.^240^ Although these evidences should be considered with caution, since patients were not precisely stratified for pain modalities, it is likely that the amygdala, by its widespread neuroanatomical connections, can contribute to nociceptive, as well as nociplastic Parkinson's disease-related pain. Lesion of the CeA, could be responsible of abnormal attribution of visceral and emotional correlates to pain, in the context of fear-anxiety network. Moreover, lesion of the Enk and NPS interneurons in the CeA and in the ITC could suppress the modulatory action on the descending anti-nociceptive system and the extinction of pain-related fear conditioning. Lesions on the BLA instead, could alter the circuitry with the mPFC and the NAc, associating pain with cognitive impairment and contributing to cognitive aspects of pain chronicization.

### Emerging role of habenula

The habenula is a phylogenetically old brain structure, preserved in all vertebrates.^241^ In mammals, it is present as bilateral small nuclei located in the postero-medial aspect of the thalamus and it can be divided into medial habenula (MHb) and lateral habenula (LHb). The habenula is involved in sensory-evoked and chronic pain induced aversion,^242,243^ in motor responses to pain and in escaping behaviors.^244^ Furthermore, it is part of the anti-reward system (negative reinforces), activated in response to aversive stimuli.^243,245,246^ Habenular nuclei, and especially the MHb are characterized by high expression of a-syn, being subject to an increased risk of a-syn aggregation during synucleinopathies.^95^ The multidirectional connectivity of the habenula makes it a possible key region in the propagation of a-syn aggregates.

Recent studies highlight the efficacy of pharmacological and electric modulation of the habenula in the treatment of depression in Parkinson's disease.^247,248^ Increased firing of the LHb has been detected in animal models of depression-pain syndrome.^244,249,250^ The LHb confers inhibitory tone to the dopaminergic projection to mPFC and NAc^251,252^ and it seems to hold a negative relay-like role in descending pain modulation from NAc to PAG.^253,254^ Also, the LHb negatively modulates the neuronal populations involved in anti-nociception, such as: the serotoninergic raphe nuclei and the noradrenergic neurons of the LC.^255-257^

The role of the habenula in pain transmission and modulation can be direct or indirect and might intervene in both emotional correlates of pain and in the modulation of the descending anti-nociceptive system. Direct projections from the spinal cord lamina I and the trigeminal spinal nucleus to the LHb have been reported in animal models.^105,241^ While, the major route to the LHb is the stria medullaris, which brings towards afferents from the basal forebrain^246,258,259^ and the lateral hypothalamus.^260^ The latter, in turn, gathers projections from the spinal laminae I and II,^258,261^ involved in the autonomic correlates of pain.^262,263^ Relevant projections to the LHb arise also from the NAc^264^ and dorsal striatum.^159,265^ More relevantly, the LHb projects to dopaminergic neurons^266^ of the VTA and the SNc and to serotonergic neurons of medial and dorsal raphe nuclei.^256,267,268^ The MHb projects predominantly to the interpeduncular nucleus via the *fasciculus retroflexus.*^245,258^

Habenula increases firing when an expected reward does not manifest, on the contrary, it decreases firing when the expected reward occurs.^269^ Accordantly, the role of the habenula in pain modulation could be encompassed in the reward-deficiency/anti-reward model (CReAM) postulated by Borsook *et al*.^171^ Pain-related reward deficiency manifests with anhedonia and decreased motivation for natural reinforces and correlates with a state of DA depletion in the shell NAc. Conversely, anti-reward entails a pain-related over recruitment of limbic structures involved in anxiety and fear, including: the CeA and BLA, the bed nucleus of the stria terminalis, the lateral tegmental noradrenergic nuclei and the raphe serotoninergic nuclei, leading to negative affective states related to pain.

The role of the habenula appears even more intriguing if we consider that chronic pain entails the same mechanisms of neuroadaptation with addiction, thanks to partly shared neural circuits.^269^ These two processes appear the opposite sides of the same spectrum, since both subtend a dysfunction in reward system characterized by abnormal reward/aversion processing, intertwined to a dysregulation in mesolimbic release of DA and in reward/aversion-related memory. Reverberant anatomical connections of the habenula seem to play a pivotal role in both the processes, realizing a feedback mechanism in the loop circuits of analgesia/hyperalgesia and reward/aversion.^253^

Disruption of the habenular circuits, which likely occurs in Parkinson's disease, might have a complex effect on the development of nociplastic Parkinson's disease-related pain and on coupling pain with depressive and anxiety states or with anhedonia. On one side habenula potentiates the negative affective states related to pain, on the other side it exerts an inhibitory effect on the circuits of reward. For this reason, disruption of the habenular loops could correlate with pain syndromes in which anhedonic and aversive states are associated and in which dysregulation of dopaminergic and serotoninergic systems might contextually occur.^270^

## Concluding remarks

Dopaminergic dysfunction is the cardinal neuroanatomical and neurophysiological alteration of Parkinson's disease. DA loss is responsible of motor symptoms and motor fluctuations, which lead in turn, to the development of musculoskeletal nociceptive pain or radicular neuropathic pain. Dopaminergic dysfunction in the nigro-striatal pathway has been also linked to a lowering in pain threshold^153^ which is a signature feature of pwPD and results often independent from the occurrence of clinically documented Parkinson's disease-related pain. Optimization of the dopaminergic treatment should be considered therefore a primary goal for the management of both acute and chronic Parkinson's disease-related pain.

Acute musculoskeletal nociceptive pain related to motor fluctuations or dyskinesias are generally responsive to levodopa treatment.^14^ Moreover, the effects of DA replacement therapy in Parkinsonian patients extends to the relief of chronic pain by rising the pain threshold. Interestingly, such an effect is specific of pwPD, suggesting that the absence of the natural timing and spacing of DA release within the basal ganglia is key to keep the gating of pain transmission at an appropriate threshold. In fact, when DA release occurs following an altered pattern (low DA levels with a non-canonical timing and spacing of DA stimulation) pain threshold is no longer effective to prevent abnormal pain perception. In the presence of physiological DA transmission, DA levels are high enough to enable the efficacy of gating circuits. In such a scenario, DA itself is not considered as an analgesic neurotransmitter and thus, increasing DA levels, does not produce analgesia in Parkinson's disease-free patients with chronic pain and an intact meso-striatal DA pathway. In pwPD, analgesic effects of DA are instead disclosed, because DA levels are below the amount needed to modulate striatal circuits in setting the appropriate pain threshold. Moreover, DA projections gather the limbic and reward circuits where DA can disclose complex and multifaced effects on pain perception. Evaluation of the associated clinical and psychological symptoms should be considered a key aspect to drive an appropriate and personalized dopaminergic treatment. In particular, the association of pain with anhedonia or apathy may support a dysfunction at the mesolimbic DA pathways. In these cases, a combined employment of D2-like agonists and antagonists should be evaluated in consideration of the *reward/anti-reward view*, in which DA can both reduce and potentiate pain perception acting on the shell or core NAc.

**Figure 2:**
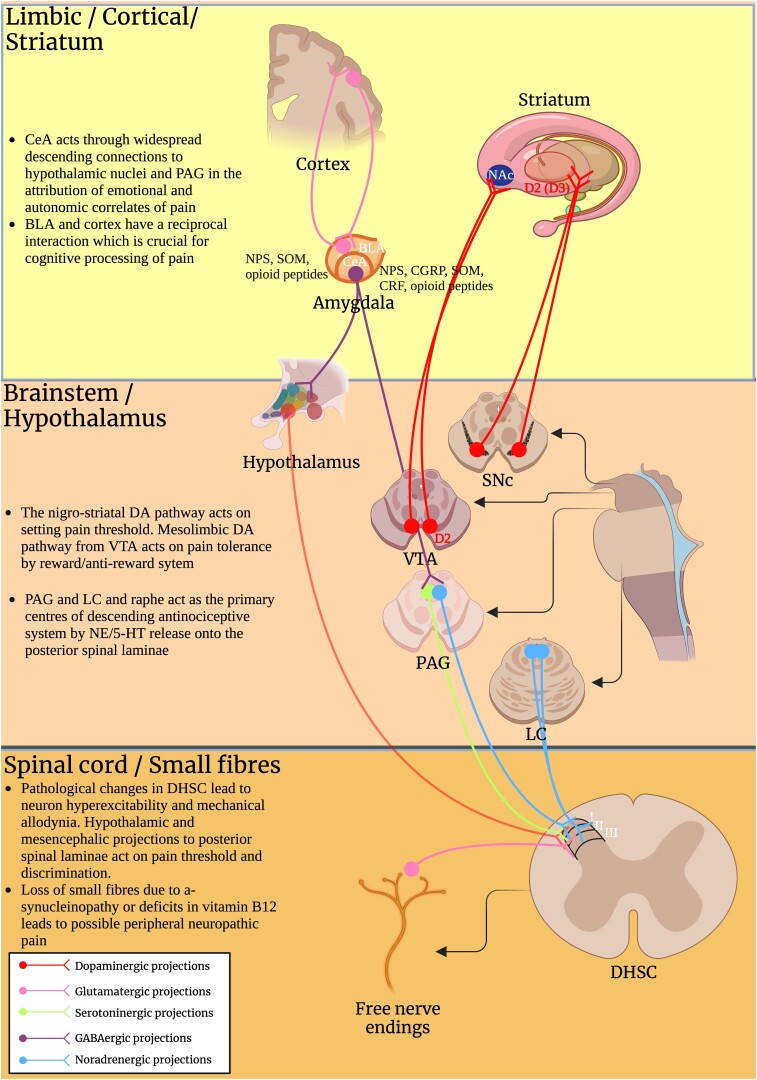
**Anatomical pathways involved in Parkinson's disease-related pain.** Schematic representation of the complex and heterogeneous anatomical network related to pain perception, processing, and modulation in the CNS. Neurotransmitters mainly involved in each circuit are highlighted in the legend at the bottom. Neuropeptides involved in amygdaloid pain processing are also reported: Somatostatin (SOM); Neuropeptide S (NPS); Calcitonine Gene-related Peptide (CGRP); Corticotropin Releasing Factor (CRF) and opioid peptides. Possible physiological and pathological implications of each neuroanatomical region in course of Parkinson's disease is reported in the didascaly. Other abbreviations include: CeA, central amygdala; PAG, Periaqueductal Gray); BLA, basolateral amygdala; NAc, nucleus accumbens; SNc, substantia nigra pars compacta; VTA, ventral tegmental area; LC, locus coeruleus; DA, dopamine; NE, norepinephrine; Serotonin (5-HT); DHSC, dorsal horn of spinal cord.

On the other hand, Wasner and Deuschl^26^ have distinguished between a DA-maintained pain and a DA independent pain (DIP), in which pain is not responsive to dopaminergic treatment. In accordance, the effect of dopaminergic agonists was found week in a systematic review on chronic pain treatment in Parkinson's disease, while safinamide, cannabinoids and opioids were associated with the highest pain reduction and selective noradrenaline transports inhibitors (SNRI) were mostly effective in sub-groups of patients with comorbidity of pain with anxiety and mood disorders.^271^ This might be explained by the heterogeneity of neurotransmitters involved in pain processing (Fig. 2) and by the widespread a-syn pathology occurring in pain network in Parkinson's disease. Neuroanatomy can help in the management of DIP, highlighting the importance of differentiated therapeutic approaches, based on the semeiology of pain and on the association of pain with other key clinical symptoms, featuring different underlying anatomical backgrounds (Table 3). Further clinical studies are requested to elucidate the neuroanatomy of pain network in Parkinson's disease. In particular, the contribution of each neuroanatomical structure needs to be validated in cohort of patients accurately stratified, towards *ad hoc* studies, in which key associated symptoms will drive the identification of the affected neuronal networks. Neuropathology and functional MRI might strongly help a neuroanatomy-based approach to Parkinson's disease-related pain. Personalized treatments should be employed, based on the anatomical and neurochemical pathways involved in each patient.

**Table 3:** Neuroanatomy-based interpretation of pain domains in Parkinson's disease

Altered pain domain	Clinical features	Comorbidities	PD-PCS	Candidate Anatomical correlates	Neurotransmitters and neuropeptides
Musculoskeletal pain	Acute or chronic pain related to motor symptoms	Wearing-off Motor fluctuations dyskinesias	Nociceptive	Discharge of peripheral nociceptors of tendons and ligaments	Dopamine Glutammate
Pain threshold and pain discrimination	Thermic and mechanical allodynia	Peripheral neuropathy of various origin, affective hypoesthesia	Peripheral and central neuropathic	a-Syn pathology in peripheral small fibres and posterior spinal cord laminae	Glutammate Substance P CGRP Met-enkephalin GABA
Endogenous anti-nociception	Increased pain persistence and intensity	Non-motor symptoms: depression, sleep disturbances	Central neuropathic	a-Syn pathology in LC, Hypothalamus and posterior spinal cord laminae	Dopamine Noradrenaline Serotonin Met- and Leu-enkephalin
Setting of pain threshold	Tonic pain—reduced pain threshold	Motor symptoms Absence of direct correlation with clinical evidence of pain	Central neuropathic and nociplastic	Pathological and functional alterations in the nigro-striatal DA pathway	Dopamine
Pain tolerance and affective pain correlates	Reduced tolerance to pain—reduced pain suppression by pleasant stimuli—altered emotional response to pain	Anhedonia Restless leg syndrome Akathisia	Nociplastic	Alterations in mesolimbic and meso-cortical DA pathways	Dopamine
Attribution of emotional and autonomic correlates to pain	Abnormal fear and anxious correlates of pain -increased autonomic response to pain	Anxiety Depression	Nociplastic	a-Syn pathology at the CentroMedian Amygdala and the LC	GABA CGRP Neuropeptide S Somatostatin Corticotropin-releasing factor Opioid peptides
Cognitive processing of pain	Chronic pain associated with cognitive symptoms (negative thinking, rigidity, anhedonia)	Cognitive impairment Behavioural symptoms	Nociplastic	a-Syn pathology at the Basolateral Amygdala	Glutammate Neuropeptide S Somatostatin Opioid Peptides
Attribution of emotional and autonomic correlates to pain	Reduced tolerance to pain associated with fear and autonomic response—increased aversive behaviours	Anhedonia—depression—anxiety	Nociplastic	Disruption of the habenular loops	Acetylcholine GABA Dopamine

## Data Availability

As a narrative review article, data sharing is not applicable to this article as no new data were created or analysed in this study.
